# A clinical phase II study with sorafenib in patients with progressive hormone-refractory prostate cancer: a study of the CESAR Central European Society for Anticancer Drug Research-EWIV

**DOI:** 10.1038/sj.bjc.6604064

**Published:** 2007-11-27

**Authors:** S Steinbild, K Mross, A Frost, R Morant, S Gillessen, C Dittrich, D Strumberg, A Hochhaus, A-R Hanauske, L Edler, I Burkholder, M Scheulen

**Affiliations:** 1 Tumor Biology Center, Albert-Ludwigs-University Freiburg; Freiburg, Germany; 2 Center for Tumor-Detection, Therapy and Prevention, St Gallen, Switzerland; 3 Kantonsspital St Gallen, Switzerland; 4 LBI-ACR VIEnna, Kaiser-Franz-Josef-Spital, and ACR-ITR VIEnna, Austria; 5 Marienhospital, Herne, Germany; 6 III Med Klinik, Med Fakultät Mannheim, Universität Heidelberg, Mannheim, Germany; 7 III Department Medicine, Asklepios Klinik, Hamburg, Germany; 8 German Cancer Research Center, Heidelberg, Germany; 9 West German Cancer Center, Essen, Germany

**Keywords:** prostate cancer, sorafenib, phase II study, hormone-refractory

## Abstract

Sorafenib is a multi-kinase inhibitor with antiangiogenic and antiproliferative activity. The activity of sorafenib in progressive hormone-refractory prostate cancer (HRPC) patients was investigated in a phase II clinical study. Progressive HRPC patients received sorafenib 400 mg bid p.o. continuously. Only patients with no prior chemotherapy, and either one-unidimensional measurable lesion according to RECIST-criteria or increasing prostate-specific antigen (PSA) values reflecting a hormone-refractory situation, were eligible for study entry. The primary study objective was the rate of progression-free survival of ⩾12 weeks (PFS12). Secondary end points were overall response, overall survival, and toxicity. Fifty-seven patients with PC were enrolled. Two patients had to be withdrawn from the set of eligible patients. According to RECIST criteria, 4 patients out of 55 evaluable patients showed stable disease (SD). According to PSA–response, we saw 11 patients with SD PSA and 2 patients were responders at 12 weeks (PFS12=17/55=31%). Among the 257 adverse events, 15 were considered drug related of maximum CTC-grade 3. Twenty-four serious adverse events occurred in 14 patients (14/55=26%). Seven of them were determined to be drug related. No treatment-related death was observed. Sorafenib has antitumour activity in HRPCP when evaluated for RECIST- and PSA-based response. Further investigation as a component of combination regimens is necessary to evaluate its definite or overall clinical benefit for HRPCP.

Sorafenib is an oral multi-kinase inhibitor that targets the Raf/MEK/ERK signalling pathway and receptor tyrosine kinases involved in tumour cell proliferation and angiogenesis (Wilhelm *et al*, 2004). *In vitro*, sorafenib inhibited b-Raf and Raf-1 (c-Raf or c-Raf-1), pro-angiogenic vascular endothelial growth factor receptor, and platelet-derived growth factor receptor (Wilhelm and Chien, 2002). Sorafenib prevented tumour cell proliferation *in vitro* and tumour growth in human xenograft models (Wilhelm *et al*, 2004).

Several tumour types have demonstrated mutations and overexpression of factors inhibited by sorafenib. The B-Raf V600E oncogene is present in 63% of melanomas (Ostman and Heldin, 2001), ⩾50% of papillary thyroid carcinomas (Lima *et al*, 2004), and 40% of sporadic colorectal cancers (Salvatore *et al*, 2004*)*. Although oncogenic Raf-1 mutations have not been detected in human cancers (Kolch *et al*, 2002), activating k-Ras mutations resulted in increased signalling through Raf-1 in 45% of patients (Downward, 2003).

Prostate cancer is an angiogenic tumour type. Several studies demonstrated expression of angiogenic factors in prostate cancer, especially the concentration of the vascular endothelial growth factor is higher in prostate cancer cells than in normal prostate tissue (Mazzucchelli *et al*, 2000). Furthermore, elevated level of basic fibroblast growth factor in the serum are evident. The expression of another angiogenic factor, thymidin phosphorylase, named platelet-derived endothelial cell growth factor, demonstrates strong correlation to the vascular density and the gleason-score in prostate cancer tissue and thus seems to be important in terms of angiogenesis in prostate cancer (Kikuno *et al*, 2003).

Single-agent sorafenib showed preliminary clinical efficacy in several solid tumours (Awada *et al*, 2005; Clark *et al*, 2005; Moore *et al*, 2005; Strumberg *et al*, 2005; Ratain *et al*, 2006). In metastatic renal cell carcinoma patients, sorafenib significantly prolonged progression-free survival (PFS) *vs* placebo in patients in whom previous therapy failed (Escudier *et al*, 2007). Sorafenib was also shown to be suitable for long-term administration because of its good safety profile (Awada *et al*, 2005; Strumberg *et al*, 2005; Abou-Alfa *et al*, 2006; Ratain *et al*, 2006).

Prostate cancer is the most common cancer in man. About 20% of all new diagnosed prostate cancers present metastatic disease and many others metastases despite treatment with surgery and radiotherapy. Treatment of metastatic prostate cancer is palliative in about 80% of men, primary androgen ablation is the treatment of choice leading to symptomatic improvement and a reduction of the PSA. Nevertheless, all patients finally get refractory to hormone treatment. The therapeutic options then include symptomatic care with analgetics, radiotherapy to the dominant sites of pain, bisphosphonates, treatment with bone-seeking isotopes, low-dose cortisone, and palliative chemotherapy. Chemotherapy can reduce PSA levels and relieve pain in some patients, but intolerability is a problem since many of the affected elderly patients exhibit other serious medical problems. Quality of life and pain control improved more frequently under therapy with mitoxantrone plus prednisone than with prednisone alone in a randomised trial. But this therapy has unfortunately no effect on survival (Tannock *et al*, 1996). Prolongation of survival has been shown recently for the combination of docetaxel plus prednisone when compared to mitoxantrone plus prednisone, which had been the previous standard chemotherapy regimen since 1996. The median survival benefit was 2.4 months in favour of docetaxel plus prednisone. Response in terms of relief from pain, PSA decrease, and increase in quality of life was also better in the docetaxel plus prednisone treatment arm (Tannock *et al*, 2004). Nevertheless, both chemotherapeutic interventions feature clinically relevant adverse effects. Therefore, further improved treatment options for hormone-refractory prostate cancer (HRPC) patients are urgently needed since only two approved cytostatic agents exist, meaning rather unsatisfying therapeutic impact for this therapeutic situation.

We conducted within the CESAR Central European Society for Anticancer Research-EWIV, a phase II study in prostate cancer patients with progressive disease (PD) while on hormone therapy, therefore judged as hormone refractory. Sorafenib was selected as study drug due to its antiangiogenic properties. Angiogenesis is a fundamental event in the progress of tumour growth and metastatic dissemination (Folkman, 1995). It was hypothesised that angiogenesis is an important event in prostate cancer as it is in other cancers and that therefore inhibition of angiogenesis should have an influence on the tumour growth.

## PATIENTS AND METHODS

### Major eligibility criteria

Patients with HRPC eligible for this study were ⩾18 years and had an Eastern Cooperative Oncology Group performance status of ⩽2 reflected by increasing PSA under antihormonal treatment with GnRH-Analoga, antiandrogenes, and hormone withdrawal. Hormone-refractory disease was defined as PSA increase of ⩾50% above nadir, on at least two successive occasions at least 1 month apart (Bubley *et al*, 1999) They had adequate bone marrow function (haemoglobin >90 g l^−1^, absolute neutrophil count ⩾1.5 × 10^9^ l^−1^, platelet count ⩾100 × 10^9^ l^−1^), adequate liver function (total bilirubin ⩽1.5 × upper limit of normal (ULN), activities of alanine amino transferase, and aspartate amino transferase ⩽2.5 × ULN), and adequate renal function (serum creatinine ⩽1.5 × ULN).

Written informed consent was obtained from all patients who participated in the study, which was conducted according to the Good Clinical Practice guidelines and the principles described in the Declaration of Helsinki and which had been approved by the Ethical Committees of participating sites.

### Treatment

Study treatment consisted of daily 800 mg sorafenib (400 mg bid) continuously given until the end of treatment. According to the study protocol, treatment was discontinued due to progression according to RECIST criteria, unacceptable drug-related toxicity, death, or withdrawal of consent. Dose reduction to 400 mg once daily and treatment delays for clinically significant drug-related haematologic or non-haematologic toxicities were allowed according to the protocol. If further dose reductions were required, the patient had to be withdrawn from the study. No other chemotherapy, hormonal therapy, or experimental medications were permitted while the patients were in the study.

### Study design

This study was designed as a one-stage non-randomised non-blinded, multicentre phase II trial to distinguish between the null hypothesis H_0_: PFS12⩽p_0_=0.20 *vs* the alternative H_1_: PFS12 ⩾p_1_=0.40. Primary end point of the analysis was the rate of PFS of at least 12 weeks (PFS12). Secondary end points were overall response, overall survival, and safety. The primary end point PFS12 is defined as ratio PFS12=*n*_TTP12_/*n*_TTP_, where *n*_TTP_ is the number of patients eligible for the evaluation of progression up to 12 weeks, and *n*_TTP12_ is the number of patients eligible for the evaluation who survived without PD (determined using RECIST- and PSA-response criteria) for at least 12 weeks. The primary efficacy variable PFS12 was planned to be analysed by a one-sided exact binomial test for proportions of testing H_0_ at the level of *α*=0.10. The sample size was planned as of 55 patients to achieve a power of 95%.

### Evaluation of response

The primary objective of the trial was to define the efficacy of sorafenib in HRPC patients. Tumour assessment was planned at baseline, thereafter at 6 and 12 weeks and from then onwards every 8 weeks until PD or death.

We considered information until 12 weeks as sufficient for RECIST, if for each target lesion at least one measurement was available at 12 weeks after start of study drug. Patients with target lesions at baseline were assessed by their target lesions. Patients with no target lesions at baseline and who developed new lesions or metastases during treatment until week 12 were classified as PD, and PSA response was not used for the response assessment. Response evaluation according to RECIST is based exclusively on differences of the sum of the longest diameters of all target lesions or on appearance of new target or non-target lesions (Therasse *et al*, 2000).

Patients with target lesions at baseline and insufficient information until 12 weeks were assessed by their PSA levels, and their PSA response was determined. Patients without target lesions at baseline and who did not show occurrence of new lesions/new metastases were evaluated for response/progression exclusively based on the presence and the development of the PSA level since start of treatment.

Classification of PSA response/progression was performed as follows: minimum requirement for PSA response was the existence of a baseline PSA level PSA_0_ determined before start of treatment, but not more than 2 weeks (Bubley *et al*, 1999) before. Furthermore, for evaluability, at least one measurement had to be available more than 1 week after the start of treatment. Otherwise, the patient was not evaluable for response. For the assignment of response, at least two further measurements were required after start of treatment: one not earlier than 1 week after start of treatment and the second at least 4 weeks after the first value. Minimum requirement for stable disease (SD) was at least one further measurement beside that of the basic value.

The PSA-response evaluation further distinguished between the magnitudes of the PSA level at baseline: patients with a baseline value below 4 ng ml^−1^ could only qualify for SD-PSA when remaining below 4 ng ml^−1^ or for PD-PSA when increasing from a value ⩽4 ng ml^−1^ for more than 100% and increasing in the absolute value by at least 5 ng ml^−1^. Patients with a baseline value above 4 ng ml^−1^ qualified for PSA response, SD-PSA, and PD-PSA according to the criteria as follows: PSA response was attained when at least two consecutive PSA levels were lower than the 50% level of the baseline level, the first at minimum 1 week after the start of treatment and a second value at least 4 weeks after that first value. Progression (PD)-PSA was attained when no decrease of PSA occurred by 12 weeks, that is: when ⩾1 week after start of treatment at least one PSA level is 100% higher than the baseline level (at least doubled between baseline and this level) without intercurrent decrease of PSA (non-decreasing course). Progression-PSA was also attained when a decrease of PSA occurred by 12 weeks, in case, if at least one PSA level is 100% higher than the nadir of PSA between baseline and this level (not non-decreasing course), and the PSA level had increased in the absolute value by at least 5 ng ml^−1^. Stable disesae-PSA was attained when neither the criteria for PD-PSA nor those for response-PSA were fulfilled. According to this approach, the 12-week response assessment was categorised as follows: response (RECIST or PSA), SD (RECIST or PSA), PD (RECIST or PSA), and early death. Early death was defined as death within 4 weeks after the start of treatment. The response according to RECIST was always the leading parameter for outcome.

### Evaluation of toxicity

Adverse events were graded according to the National Cancer Institute Common Terminology Criteria v3.0. A thorough safety evaluation was performed with physical examination, analyses of haematology, and biochemistry data in 2- and 4-week intervals during treatment, respectively. For each symptom in each patient, the maximum CTC grade was determined and counted according to its annotated relation to treatment.

### Statistical analysis

The characteristics of the study population and the outcome of the primary and secondary end points were presented using descriptive statistical methods. The exact binomial test was used to test the one-sample, one-sided study hypothesis and Clopper–Pearson confidence intervals (CIs) were calculated for frequencies.

Survival curves were calculated and graphically presented using the Kaplan–Meier method for censored failure time data. Confidence intervals were calculated for survival, using the Greenwood formula and for medians, using the method of Brookmeyer and Crowley.

Patient's overall survival time was defined as the interval from date of the first intake of study drug to death (from any cause) or to last follow-up information for living patients (censored observation).

Patient's PFS was defined as the interval from date of the first intake of study drug to the date of progression or death whichever occurs first or to last follow-up information for living patients (censored observation).

Statistical analyses were performed using the statistical packages SAS for Windows Version 9.1 (SAS Institute Inc., Cary, NC, USA) and R Version 2.1.1 (http://www.r-project.org).

## RESULTS

### Patient population and tumour characteristics

Between August 2004 and June 2005, a total of 57 patients were recruited for treatment into the study by seven institutions. Owing to study-relevant protocol deviations, two patients were excluded from the efficacy set: one patient received chemotherapy before study entry, and the other patient withdrew the consent before start of the study. Median age of the 55 eligible patients was 70 years (range 52–82 years). Predominant ECOG performance status was 1 (*n*=29; 53%); 25 patients (46%) had status 0 and one (2%) had status 2. The majority of the recruited patients (*n*=41; 75%) had both locally advanced and metastatic prostate cancer. All patients were pretreated with antihormonal therapy, alone or in different combinations: 17 patients had only antihormonal therapy, 7 patients had antihormonal and radiotherapy, 7 patients had antihormonal and surgical therapy, and 24 patients had antihormonal, radio- and surgical therapy. Locally advanced cancer was seen in seven patients (13%) and metastatic prostate cancer in seven patients (13%). A summary of baseline patient and tumour characteristics is displayed in Table 1. At baseline, a total of 80 lesions (measurable and non-measurable) were recorded in 34 patients. The most frequent sites of distant metastases were bone (43/80=54%), lymph nodes (27/80=34%), liver (4/80=5%), and lung (3/80=4%). Twenty-one patients had combinations of multiple lesions. The most frequent localisations were bone (7/21=33%), lymph nodes and bone (6/21=29%), bone and other (2/21=10%), and multiple lymph nodes (2/21=10%).

### Primary efficacy

The categorisation according to RECIST resulted in no complete responses, no partial responses, four patients with SD, four patients with PD, and no early deaths. Fourty-seven patients could not be evaluated with regard to RECIST because they did not have measurable lesions. But all of those 47 patients were evaluable for PSA response so that in a second step, PSA response was evaluated as described in the section, evaluation of response.

The categorisation according to PSA showed 2 response-PSA, 11 SD-PSA, 21 PD-PSA, and 13 unknown (PSA). In the last case, PSA was not assessable or there were insufficient data.

The outcome of the combined 12-week response was (see Table 2): 2 responder PSA, 15 SD (4 RECIST and 11 PSA), 25 PD (4 RECIST and 21 PSA), no early death, and 13 unknown (PSA). On the basis of five SD and two responders until 12 weeks, the PFS12 rate was 31% (17/55, 95% CI: 19–45%). This was sufficient to reject the study null hypothesis H_0_: PFS12⩽p_0_=0.20 with a statistical significance of 0.05 (*P*=0.037). Therefore, this phase II trial concludes in a significant effect and demonstrates activity with respect to its primary objective of the PSF12 rate.

### Survival

**Figure 1:**
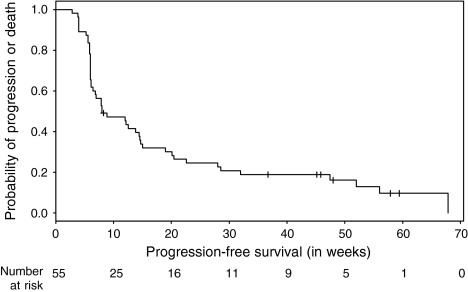
Probability of PFS (*n*=55).

**Figure 2:**
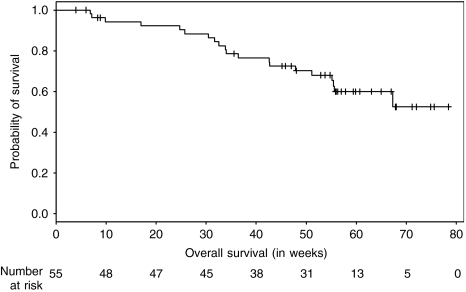
Probability of overall survival (*n*=55).

The median PFS was 8 weeks (95% CI: 6.4–14.7 weeks) and the 1 year PFS rate was 13% (95% CI: 6–28%). The Kaplan–Meier plot for PFS in all patients is shown in Figure 1. At the time of analysis, 20 out of 55 patients (36%) had died, but the median survival time was not reached yet. The 1-year overall survival rate was estimated as 68% (95% CI: 56–82%). Figure 2 shows the Kaplan–Meier plot for overall survival.

### Drug safety

All 55 patients were evaluable for safety. In total, 257 different adverse events (AEs) (referring to maximum CTC grade per patient) were documented including 37 (14%) AEs with maximum CTC grade 3. The AEs of CTC grade 4 were not observed. Table 3 shows maximal CTC grade per patient and symptom (only for symptoms occurring in five or more patients) in relation to study drug. Most frequently occurring drug-related AEs in the form of clinical symptoms were fatigue, dermatological side effects, and diarrhoea. Fifteen drug-related events of maximum CTC grade 3 were recorded in 15 patients (*n*=4 definite, *n*=6 probable, and *n*=5 possible). The most common drug-related AEs of maximum CTC grade 3 were hypertension, rash in the form of acneiform erruptions, desquamation of the skin, fatigue, and constipation. Twenty-four serious adverse events occurred in 14 (26%) patients. Seven serious adverse events in six patients were assessed to be related to the study medication (*n*=3 probable, *n*=4 possible). No treatment-related deaths occurred.

The most frequently occurring AEs based on clinical chemistries of maximum CTC grade 3 were increase of the activities of alkaline phosphatase (16%), ASAT (4%), hypophosphataemia (7%), lipase (4%), hypocalcaemia (7%), PT-INR (4%), and anaemia (4%). In only one patient, we saw decreased platelets of CTC grade 3 and a lymphopaenia of CTC grade 3. No CTC grade 4 haematological toxicity occurred.

At least one dose reduction occurred in 9 out of 55 (16%) evaluable patients. In four patients (7%), therapy was interrupted for at least one time interval until treatment-related toxicity had resolved to grade 2, except for skin toxicity, which had to resolve to grades 0–1. Three patients received both dose reduction and interruption of therapy.

## DISCUSSION

This phase II trial was performed to evaluate the activity and safety of sorafenib in patients with HRPC without prior systemic chemotherapy. Fifteen out of 55 eligible patients showed SD (4 RECIST and 11 PSA) and two patients were PSA responder (PFS12=17/55=31%). Therefore, this phase II trial concludes a significant effect and demonstrates activity with respect to primary end point PFS12. The treatment in the form of long-term continuous oral administration exerted only mild toxicity.

Given the difficulties to assess tumour response and given the uncertainty about the predictive value of response for clinical benefit, a careful interpretation of the results is indicated. Therefore, we conclude that sorafenib 400 mg bid has only moderate anticancer activity in HRPC patients. It has a mild-toxicity profile and is well tolerated. Especially with regard to quality of life, patients have a benefit, since conventional chemotherapy for prostate cancer causes side effects and is sometimes not well tolerated, often observed in elderly patients. Nevertheless, single-agent therapy displays only limited impact.

Similar results with single-agent targeted therapy for prostate cancer patients were demonstrated. In a phase II study with sorafenib 400 mg bid in 16 patients with HRPC, one patient had a confirmed PSA-response and four patients had post-treatment PSA decline without any other immediate therapy. Treatment was generally well tolerated and according to the authors, the results indicate a potential detrimental effect and a positive delayed effect on PSA production and secretion (Chi *et al*, 2005).

Another phase II trial with pertuzumab, a humanised monoclonal antibody, was conducted for patients with HRPC in progression after taxane therapy. Pertuzumab represents a new class of targeted anticancer agents that inhibit human epidermal growth factor receptor dimerisation. The human epidermal growth factor receptor family of receptors dimerises and activates intracellular signalling pathways, leading to cellular growth, proliferation, and survival. Preclinical studies demonstrated that inhibition of ligand-dependent heterodimerisation with pertuzumab effectively inhibits tumour growth and diminishes mitogen-activated protein kinase and phosphatidyl-inositol 3-kinase activity in both androgen-dependent and -independent prostate cancer xenograft models. In 30 assessable patients, no objective responses were seen, but four patients had SD for at least 23 weeks and one had SD for 36 weeks. The author underlined the clinical benefit for the patients (Agus *et al*, 2007).

Since docetaxel has become the status of a standard therapy in HRPC patients, there are efforts to improve the efficacy of docetaxel-based chemotherapy for HRPC. This includes combining docetaxel with other agents with novel mechanisms of action, such as atrasentan (George *et al*, 2005), thalidomide (Dahut *et al*, 2004), bevacizumab (Berry and Eisenberger, 2005), and DN-101 (Beer *et al*, 2007). The combination of docetaxel plus sorafenib may represent a contribution to these efforts on the basis that this drug combination was tested within a phase I study and showed clinical activity in patients with advanced, refractory solid tumours (Awada *et al*, 2007). Interestingly, the combination showed an increase of docetaxel AUC_0–24_ and *C*_max_ in a dose-independent manner_._ The most common side effect was dermatologic toxicity that led to a dose reduction or interruption of sorafenib in 60% of patients. The authors suggest a dosing schedule of docetaxel and sorafenib 400 mg bid since cutaneous toxicity is not a life-threatening side effect. In case of significant dermatological toxicity, sorafenib dose should be reduced to 200 mg bid. Such an approach should be evaluated in a phase II study in HRPC.

## Figures and Tables

**Table 1:** Patient and tumor characteristics (*n*=55 evaluable patients)

**Period of recruitment**	**October 2004–June 2005**	
**Age (median, range)**	**70 (52–82) years**	
	***n***	**%**
*Performance status*		
0	25	46
1	29	53
2	1	2
		
*Tumour characteristics*		
Advanced	7	13
Metastatic	7	13
Advanced and metastatic	41	75
		
*Grade of differentiation*		
G1	1	2
G2	17	31
G3	21	38
GX	16	29

**Table 2:** RECIST and PSA-based response evaluation

**Response category**	**RECIST**	**PSA**	**Total (*n*=55)**
Response	0	2	2
Stable disease (SD)	4	11	15
Progressive disease (PD)	4	21	25
Early death	0	0	0
Unknown	—a	13	13

a PSA response was evaluated only in patients of the RECIST-unknown category.

**Table 3:** Severest adverse event per patient and relation to study drug (only symptoms occurring in five or more patients)

**CTC-Symptom**	**Total (drug related)**	**CTC grade 1 (drug related)**	**CTC grade 2 (drug related)**	**CTC grade 3 (drug related)**
Fatigue	21 (13)	12 (5)	7 (6)	2 (2)
Pain	19 (3)	7 (3)	5 (0)	7 (0)
Skin	18 (16)	12 (11)	4 (3)	2 (2)
Diarrhoea	10 (10)	7 (7)	2 (2)	1 (1)
Neurologic	10 (6)	8 (6)	2 (0)	0
Hypertension	9 (9)	1 (1)	5 (5)	3 (3)
Infection	9 (0)	4 (0)	4 (0)	1 (0)
Constitutional symptoms	8 (5)	4 (3)	3 (2)	1 (0)
Gastrointestinal symptoms	8 (7)	7 (6)	1 (1)	0
Nausea	8 (6)	6 (5)	1 (1)	1 (0)
Anorexia	7 (4)	5 (4)	2 (0)	0
Constipation	7 (3)	4 (1)	1 (0)	2 (2)
Renal/Genitourinary symptoms	7 (0)	5 (0)	1 (0)	1 (0)
Hand-foot skin reaction	6 (6)	4 (4)	1 (1)	1 (1)
Weight loss	6 (0)	6 (0)	0	0
Hair loss	5 (5)	4 (4)	1 (1)	0
Mood alteration	5 (1)	4 (1)	1 (0)	0
Rash/desquamation	5 (5)	3 (3)	0	2 (2)
